# Ethanol Extract of *Sesamum indicum* Linn. Inhibits Fc*ε*RI-Mediated Allergic Reaction via Regulation of Lyn/Syk and Fyn Signaling Pathways in Rat Basophilic Leukemic RBL-2H3 Mast Cells

**DOI:** 10.1155/2019/5914396

**Published:** 2019-10-10

**Authors:** Hyun Ju Do, Tae Woo Oh, Kwang-Il Park

**Affiliations:** Korean Medicine (KM)-Application Center, Korea Institute of Oriental Medicine (KIOM), 70 Cheomdan-ro, Dong-gu, Daegu 41062, Republic of Korea

## Abstract

This study is aimed at determining whether Sesamum indicum Linn. beneficially influences Fc*ε*RI-mediated allergic reactions in RBL-2H3 mast cells; it is also aimed at further investigating Lyn/Fyn and Syk signaling pathways. To examine the antiallergic effect of *Sesamum indicum* Linn. extract (SIE), we treated antigen/immunoglobulin E- (IgE-) sensitized mast cells with extracts of various concentrations. We examined the degranulation release and concentrations of inflammatory mediators. Additionally, the expressions of genes involved in the Fc*ε*RI and arachidonate signaling pathways were examined. SIE inhibited the degranulation and secretion of inflammatory mediators in antigen/IgE-sensitized mast cells. SIE reduced the expressions of Fc*ε*RI signaling-related genes, such as Syk, Lyn, and Fyn, and the phosphorylation of extracellular signal-regulated kinase in antigen/IgE-sensitized mast cells. Additionally, in late allergic responses, SIE reduced PGD_2_ release and COX-2 and cPLA2 phosphorylation expression in Fc*ε*RI-mediated mast cell activation. Lastly, 250–500 mg/kg SIE significantly attenuated the Ag/IgE-induced passive cutaneous anaphylaxis (PCA) reaction in mice. The potent effect of SIE on RBL-2H3 mast cell activation indicates that the extract could potentially be used as a novel inhibitor against allergic reactions.

## 1. Introduction

Mast cells are widely distributed across the human body and are present in various regions, including the skin, respiratory, and digestive systems [1]. These cells generate a large number of mediators that induce inflammation and immune responses to various external stimuli [1, 2]. Thus, they are crucial in natural and adaptive immune responses. They also play a critical role in the onset and progress of allergic inflammatory diseases, such as asthma, psoriasis, and arthritis. Mast cells are also important in the progression of allergy and anaphylaxis [3]. RBL-2H3 mast cells exhibit the properties of mucosal mast cells, and their most distinctive type comprises several cellular granules and crystallized chemical mediators [4]. Once allergic antigens are cross-linked with immunoglobulin E (IgE) that is bound to the high-affinity IgE receptor (Fc*ε*RI) on the cell surface, histamine and *β*-hexosaminidase are released [5], which activates RBL-2H3 cells and causes their degranulation. Thus, RBL-2H3 cells have been generally used to study IgE-Fc*ε*RI interaction and signaling pathways, such as the degranulation and binding of IgE to Fc*ε*RI receptors [5, 6]. Similarly, we have used RBL-2H3 cells as a model cell line to assess the inhibitory effect of Lyn/Syk and Fyn against Fc*ε*RI-mediated mast cell activation.

*Sesamum indicum* Linn. (SI) is one of the most important seed crops and traditional health food in Asian countries. Most pharmacological studies on S. indicum seeds have reported hypoglycemic effects in genetic diabetes, antitumor effects, antiestrogenic activity, positive effects on Parkinson's disease patients, antihypertensive effects, and increased vitamin E levels without the use of vitamin E supplements. In addition, aqueous defatted seed extracts from S. indicum have already been demonstrated to exhibit hypoglycemic and hypolipidemic activities. Thus, SI has been shown to have fat-lowering effects in hypercholesterolemic rats; it reduces weight gain caused by cholesterol intake and reinforces the antioxidant defense system in the body [7]. In addition, SI has been reported to inhibit liver damage and oxidative stress in septic mice [8]. SI contains high amounts of plant lignans, including sesamin, sesamolin, and sesaminol glucosides. Sesamin, the major fat-soluble lignan in sesame seeds, influences lipid metabolism and has antihypertensive and anticancer properties [9, 10]. However, only a limited number of studies have examined antiallergenic properties of extracts from sesame seeds.

The present study investigated the antiallergic activities of the *Sesamum indicum* Linn. extract (SIE) against Fc*ε*RI-mediated allergic reactions in mast cells and the mechanism by which SIE suppresses these allergic reactions. The results of this study would be important in the advancement of plant medicines against allergic diseases.

## 2. Material and Methods

### 2.1. SIE Preparation

The samples of *Sesamum indicum* Linn. (SI) were obtained as dried herbs from Yeongcheon Oriental Herbal Market (Yeongcheon, South Korea) and were authenticated by the Korean Medicine Application Center, Korea Institute of Oriental Medicine. SI (50 g) was extracted using 70% ethanol at 40°C for 24 hr in a shaking incubator. Subsequently, the extract was filtered using a 150 *μ*m filter paper and concentrated in a rotary vacuum evaporator (Buchi, Tokyo, Japan). Samples were freeze-dried and stored at -20°C before use. Sample acquisition was 5.4 g, and the yield was 18.5%. The lyophilized powder was resuspended in distilled water, centrifuged at 10,000×g for 15 min, and filtered through a 0.2 *μ*m sterile filter to prepare the SIE. The HPLC analysis method and results are given in Supplementary Materials ([Supplementary-material supplementary-material-1]).

### 2.2. Cell Culture and Drug Treatment

Rat RBL-2H3 mast cells were cultured in Minimum Essential Medium-*α* (MEM-*α*) containing 10% heat-inactivated fetal bovine serum (FBS) and 1% antibiotics (100,000 U/L penicillamine and 100 mg/L streptomycin) at 37°C in humidified 5% CO_2_ conditions. The cells were seeded onto a 6-well plate (3 × 10^5^ cells/well) or 96-well plate (2 × 10^4^ cells/well) for 24 hr. At day 2 postconfluence, the medium was changed to MEM-*α* (10% FBS and 1% antibiotics) containing dinitrophenyl (DNP)-IgE (0.1 *μ*g/mL) for 16 hr. The medium was replaced with serum-free MEM-*α* (1% FBS and 1% antibiotics). The cells were pretreated with SIE (100, 300, and 500 *μ*g/mL) or 100 nM dexamethasone before DNP-human serum albumin (0.1 *μ*g/mL) treatment for 10 min or 4 hr.

### 2.3. Cell Viability

The RBL-2H3 mast cells were treated with various concentrations of SIE for 1 hr. To determine cell cytotoxicity, the cells were incubated with 0.5 mg/mL of MTT reagent for 40 min at 37°C. The medium was discarded, and dimethyl sulfoxide was added to the cells for 5 min. Absorbance readings at 570 nm were obtained using a microplate reader.

### 2.4. N-Acetyl-*β*-d-glucosaminidase (*β*-Hexosaminidase) Release Assay

RBL-2H3 mast cells were seeded into a 96-well plate and were pretreated with drug. Subsequently, 25 *μ*L of 4-methyl-umbellyferyl-N-acetyl-*β*-d-glucosaminidase (10 mM) was added to the supernatant of the sensitized cells with IgE for 1 hour at 37°C. To stop the reaction, the solution was mixed with sodium carbonate buffer (0.1 M, pH 10.0), and the absorbance was measured at 405 nm on a microplate reader.

### 2.5. Measurement of Inflammatory Mediators

The cells were pretreated with Sesamum indicum Linn. extract (SIE; 100, 300, and 500 *μ*g/mL) or 100 nM dexamethasone before DNP-human serum albumin (0.1 *μ*g/mL) treatment for 4 hr. The concentrations of TNF-*α* (R&D Systems, MN, USA), IL-4 (eBioscience, CA, USA), IL-6 (Thermo Fisher Scientific, MA, USA), histamine (ENZO, NY, USA), and PGD2 (Cayman, MI, USA) in the cell culture media were measured according to the manufacturer's instructions.

### 2.6. Immunoblot Analysis

The RBL-2H3 mast cells were sensitized with IgE for 10 min or 4 hr. Total proteins were extracted using RIPA buffer (Merck Millipore, Darmstadt, Germany) containing a protease and phosphatase inhibitor cocktail (Roche, Basel, Switzerland). Proteins were quantified using the bicinchoninic acid assay and were then separated by 10% sodium dodecyl sulfate polyacrylamide gel electrophoresis and transferred onto an activated polyvinylidene difluoride membrane for 100 min. The blots were blocked with 5% BSA and incubated with primary antibodies (1 : 1000) at 4°C overnight and then incubated with horseradish peroxidase-conjugated secondary antibodies for 1 hr at room temperature. Protein expressions were detected using a western blot detection kit (Thermo Fisher Scientific, MA, USA) and ChemiDoc™ Touch Imaging System (Bio-Rad, CA, USA).

### 2.7. Animals

Male ICR mice, 5 weeks of age, were randomly assigned to five groups after 1 week adaptation period: control group (CTL, *n* = 5), Ag/IgE group (Ag/IgE, *n* = 5), Ag/IgE treated with 10 mg/kg dexamethasone group (Dex, *n* = 5), Ag/IgE treated with 250 mg/kg SIE group (SIE 250, *n* = 5), and Ag/IgE treated with 500 mg/kg SIE group (SIE 500, *n* = 5). SIE was prepared in saline, and CTL and Ag/IgE groups received equivalent volumes of saline. All experiments were approved by the Committee on Animal Experimentation and Ethics of KIOM.

### 2.8. Passive Cutaneous Anaphylaxis (PCA) in Mice

At day 1, anti-DNP-IgE (4 *μ*g/mL) antibody was subcutaneously injected into the ears of mice. At day 2, IgE-sensitized mice were administered oral SIE (250 or 500 mg/kg) or dexamethasone (10 mg/kg). One hour later, DNP-HSA (300 *μ*g/mL) containing 1% Evans blue was injected into the tail veins. One hour later, the mice were sacrificed using CO_2_ and tissues from the treated ears were obtained. The Evans blue dye was removed by the ear tissue, which were then incubated with 0.4 mL formamide at 63°C for 16 hr. Absorbance at 620 nm wavelength was measured using a microplate reader.

### 2.9. Histology

For histological analysis, ear tissues were fixed in 100 mL of 4% paraformaldehyde in 0.1 M phosphate buffer (pH 7.4). 5 *μ*m thick sections of the paraffin-embedded ear tissue blocks were cut on a cryoultramicrotome (Leica, Wetzlar, Germany), mounted on positively charged glass slides, and dried in an oven at 60°C for 30 min. The sections were deparaffinized in xylene and then rehydrated in graded ethanol and water. Endogenous peroxidase was blocked by incubation in 3% (*v*/*v*) hydrogen peroxide for 10 min. Nonspecific endogenous protein binding was blocked using 1% bovine serum albumin (BSA). Sections were counterstained with hematoxylin-eosin (H&E) staining or toluidine blue. The specimens were mounted with Permount (Fisher, Fair Lawn, NJ), and images were captured using a Nikon fluorescence microscope equipped with NIS-Elements BR 4.50 software (Nikon, Tokyo, Japan). Histopathological changes of eosinophils in H&E-stained tissues and mast cells in toluidine blue-stained tissues were counted in three different parts by blind observed under microscope with 100x and 400x magnification.

### 2.10. Statistical Analysis

Data were expressed as mean ± S.E. from three independent experiments. One-way analysis of variance and Bonferroni's post hoc test were used to determine the statistical significance of differences between each treated group and the negative control (IgE group) using the GraphPad Prism software (GraphPad Prism software Inc., version 5.02, La Jolla, CA, USA). ^∗^*p* < 0.05, ^∗∗^*p* < 0.005, and ^∗∗∗^*p* < 0.0005 were considered statistically significant.

## 3. Results

### 3.1. Effect of SIE on IgE-Induced Degranulation and Inflammatory Mediators in RBL-2H3 Mast Cells

**Figure 1:**
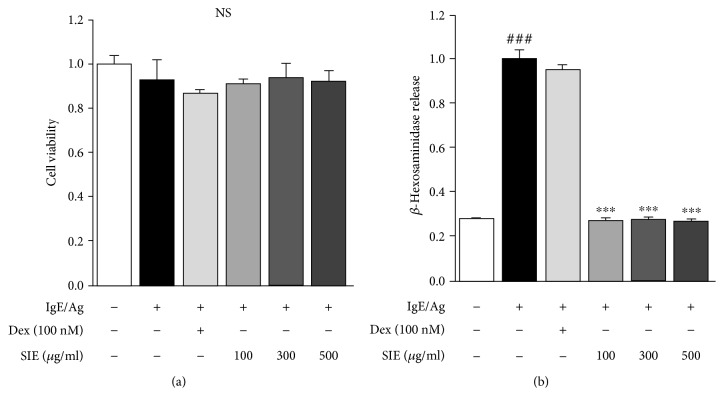
Effects of SIE on (a) cell viability and (b) *β*-hexosaminidase release in the RBL-2H3 mast cells. The results are expressed as means ± S.E. of at least three independent experimental results, tested by analysis of variance using Bonferroni's post hoc test; ^###^*p* < 0.0005 considered as indicative of a significant differences versus the control group and ^∗∗∗^*p* < 0.0005 as indicative of significant differences versus the IgE/Ag-treated group. NS: nonsignificant at the 0.05 probability level.

**Figure 2:**
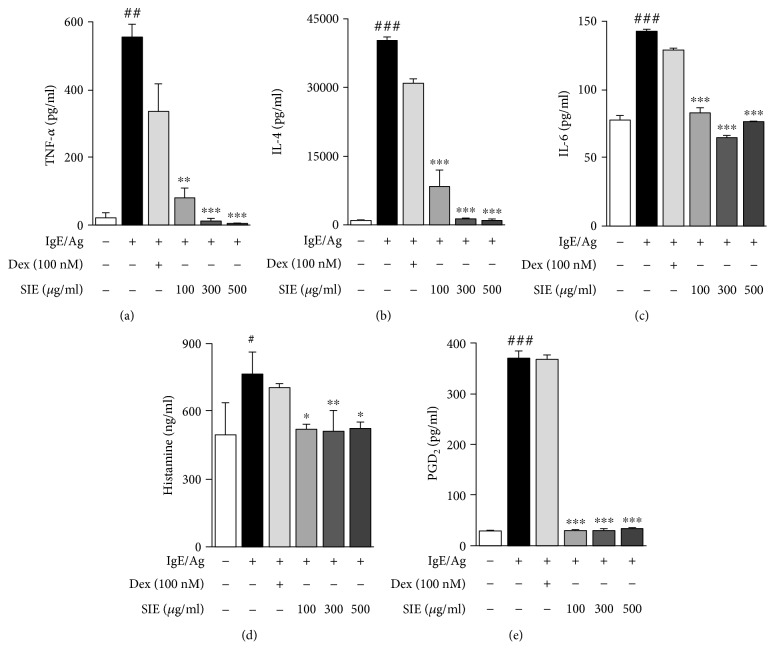
Effects of SIE on proinflammatory cytokines, including (a) TNF-*α*, (b) IL-4, (c) IL-6, (d) histamine, and (e) PGD_2_ in the RBL-2H3 mast cells. The results are expressed as means ± S.E. of at least three independent experimental results, tested by analysis of variance using Bonferroni's post hoc test; ^#^*p* < 0.05, ^##^*p* < 0.005, and ^###^*p* < 0.0005 considered as indicative of a significant differences versus the control group and ^∗^*p* < 0.05, ^∗∗^*p* < 0.005, and ^∗∗∗^*p* < 0.0005 as indicative of significant differences versus the IgE/Ag-treated group. NS: nonsignificant at the 0.05 probability level.

To determine the cell viability rate of SIE treatment on IgE-induced RBL-2H3 mast cells, we performed the MTT assay and found that SIE did not adversely affect cell viability at concentrations of 100, 300, and 500 *μ*g/mL (Figure 1(a)). *β*-Hexosaminidase release as a marker of degranulation in mast cells is used as a good indicator of the degree of allergic reaction [11]. At the initial screening stage, the experiment was conducted using low concentrations (1–10 *μ*g/mL); however, they were not effective. Consequently, we conducted the study using relatively high concentrations (100–500 *μ*g/mL), which are the concentrations that have been applied in previous studies [12]. As shown in Figure 1(b), SIE significantly decreased the *β*-hexosaminidase release of mast cells at all doses ranging from 100 to 500 *μ*g/mL. SIE treatment decreased the TNF-*α* and IL-4 concentrations in the IgE-sensitized RBL-2H3 cells in a concentration-dependent manner (Figures 2(a) and 2(b)). Similarly, IL-6 levels were significantly lower in the SIE treatment group than in the Ag/IgE-mediated RBL-2H3 mast cells (Figure 2(c)).

### 3.2. Effects of SIE on the Fc*ε*RI Signaling Pathway in RBL-2H3 Mast Cells

**Figure 3:**
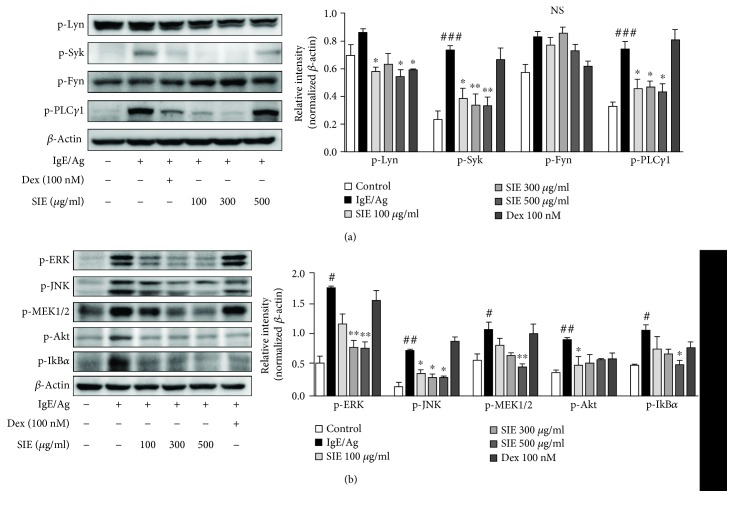
Effects of SIE on the Fc*ε*RI signaling pathways in RBL-2H3 mast cells. In the Fc*ε*RI signaling pathway, immunoblot analysis was performed by using (a) anti-p-Syk, p-Lyn, p-Fyn, and p-PLC*γ*1 and (b) p-ERK, p-JNK, p-MEK1/2, p-Akt, and p-IkB*α* antibodies. Results are expressed as means ± S.E. of at least five independent experimental results, tested by analysis of variance using Bonferroni's post hoc test; ^#^*p* < 0.05 and ^###^*p* < 0.0005 considered as indicative of significant differences versus the control group and ^∗^*p* < 0.05, ^∗∗^*p* < 0.005, and ^∗∗∗^*p* < 0.0005 as indicative of significant differences versus the IgE/Ag-treated group. NS: nonsignificant at the 0.05 probability level.

As shown in Figure 2(d), SIE inhibited Ag/IgE-mediated histamine release on RBL-2H3 mast cells. SIE reduced the phosphorylated level of Lyn and Syk protein in the Fc*ε*RI signaling cascade. However, SIE had no effect on phosphorylation of Fyn. Additionally, SIE dramatically decreased the phosphorylation of phospholipase C*γ*1 (PLC*γ*1), which is involved in the degranulation process of mast cells (Figure 3(a)). Furthermore, SIE markedly reduced the phosphorylation of mitogen-activated protein kinases (MAPKs), such as ERK, JNK, and MEK1/2. The phosphorylation of ERK, JNK, and MEK1/2 was reduced by SIE in a dose-dependent manner. Similarly, AKT and IkB*α* phosphorylation was also reduced, which demonstrated that SIE activated the Fc*ε*RI signaling pathway in Ag/IgE-activated RBL-2H3 cells (Figure 3(b)).

### 3.3. Effects of SIE on the Arachidonate Signaling Pathway in RBL-2H3 Mast Cells

**Figure 4:**
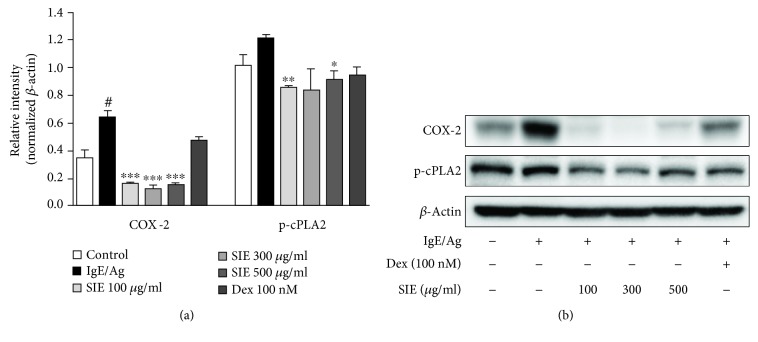
(a, b) Effects of SIE on the arachidonate signaling pathways in the RBL-2H3 mast cells. In the arachidonate signaling pathway, immunoblot analysis was performed by using anti-COX-2 and p-cPLA2 antibodies. *β*-Actin was used as a protein loading control. Results are expressed as means ± S.E. of at least five independent experimental results, tested by analysis of variance using Bonferroni's post hoc test; ^#^*p* < 0.05 considered as indicative of significant differences versus the control group and ^∗^*p* < 0.05, ^∗∗^*p* < 0.005, and ^∗∗∗^*p* < 0.0005 as indicative of significant differences versus the IgE/Ag-treated group.

Next, we observed the effect of SIE on arachidonate cascade activation, such as on the concentration of PGD2 and expressions of COX-2 and p-cPLA2 implicated in Fc*ε*RI receptor activation [13]. The concentrations of PGD2 significantly decreased at all concentrations (Figure 2(e)). We tested the antiallergic effects of SIE on the arachidonate signaling pathway, and the activations of COX-2 and p-cPLA2 were examined in Ag/IgE-activated RBL-2H3 mast cells. As shown in Figures 4(a) and 4(b), we observed a decreasing trend in the COX-2 and p-cPLA2 protein levels upon treatment with SIE.

### 3.4. Effect of SIE on Allergic Responses in the PCA Model

**Figure 5:**
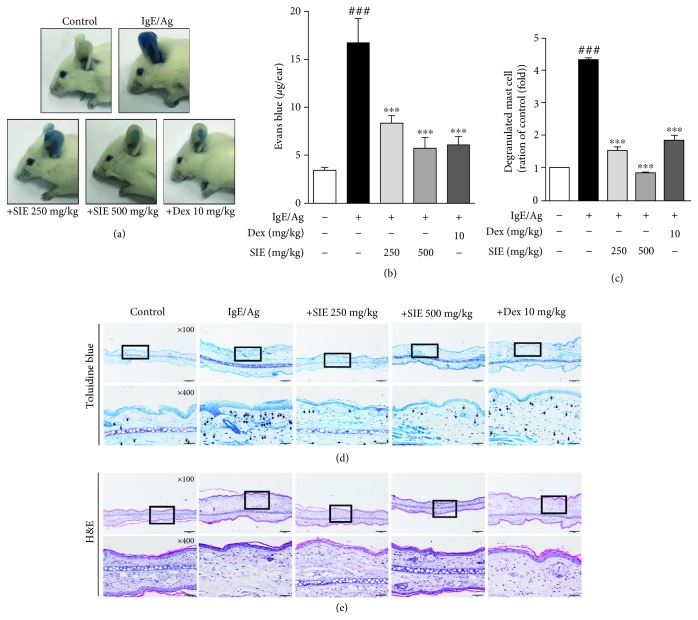
Effect of SIE on the Ag/IgE-induced passive cutaneous anaphylaxis model. ICR mice were intradermally administered DNP-IgE (4 *μ*g/mL) via the ear for 24 hr and were then were orally administered SIE (250 and/or 500 mg/mL) and dexamethasone (10 mg/mL). One hour later, DNP-HSA (300 *μ*g/mL) containing 1% Evans blue was intravenously injected into their tail veins for 1 hr. (a, b) The extravasated dye in the ears was analyzed using the procedure described in Material and Methods. (c) The average numbers of mast cells per field. Paraffin-embedded skin sections were prepared and stained with (d) toluidine blue and (e) H&E staining. Bars = 100 *μ*m. Results are expressed as means ± S.E. of at least five independent experimental results that were tested by analysis of variance with Bonferroni's post hoc testing; ^###^*p* < 0.0005 versus the control group; ^∗∗∗^*p* < 0.0005 versus the IgE/Ag-treated group.

The concentration of Evans blue significantly increased from 3.43 ± 0.28 *μ*g/ear in the CTL group to 16.70 ± 2.51 *μ*g/ear in the Ag/IgE group with the PCA reaction (*p* < 0.0005). Concentrations of Evans blue were significantly lower in the SIE 250 group (8.33 ± 0.77 *μ*g/ear, *p* < 0.0005), the SIE 500 group (5.71 ± 1.11 *μ*g/ear, *p* < 0.0005), and the Dex group (6.08 ± 0.85 *μ*g/ear, *p* < 0.0005) (Figures 5(a) and 5(b)). To evaluate the effect of SIE extract on inflammatory response, we investigated the effects of SIE extract on ear inflammation; histological changes in ear tissues were observed by H&E and toluidine blue staining (Figures 5(d) and 5(e)). H&E staining confirmed the infiltration of inflammatory cells in the PCA-induced mice. The administration of SIE at doses of 250 and 500 mg/kg inhibited edema and inflammatory response by histopathological and morphometric analysis of ear sections. Furthermore, the administration of SIE at a dose of 500 mg/kg significantly decreased the numbers of infiltrating eosinophils. Toluidine blue staining confirmed that mast cells were elevated in the control and IgE/Ag groups. Application of SIE extract treatment group reduced the mast cell numbers, and application of Dex group significantly inhibited mast cell infiltration into ear tissues (Figure 5(c)). These data indicated that SIE may inhibit IgE mediated skin pruritus by down-regulating mast cell activation.

## 4. Discussion

Mast cells are key effector cells in Fc*ε*RI-mediated allergic responses and can secrete inflammatory mediators, cytokines, growth factors, chemokines, leukotrienes, and hypersensitive factors that activate the immune system upon stimulation by an Fc*ε*RI-mediated allergic reaction [14, 15]. In the present study, we found that SIE inhibited the degranulation process and production of proinflammatory mediators, such as TNF-*α*, IL-4, IL-6, histamine, and PGD2, on Fc*ε*RI-mediated allergic reactions in RBL-2H3 mast cells. To elucidate the underlying mechanism, we evaluated the control of antiallergic effects and the expression of Fc*ε*RI signaling-related genes known to influence the concentrations of proinflammatory mediators.

Fc*ε*RI on the surface of mast cells consists of *α-*, *β-*, and *γ*-subunits. When allergens are cross-linked with IgE, the receptor is phosphorylated by Src kinase, which is bound to the *β*-subunit, to initiate intracellular signaling [16]. In addition, the *β*- and *γ*-subunits phosphorylate the immunoreceptor tyrosine-based activation motif (ITAM) via Lyn kinase, which is bound to the receptor [17]. The phosphorylated ITAM provides a binding site for Syk kinase that plays an important role in cell activation; ITAM activates Syk kinase and then induces activation of the lower signaling substance [18]. In the early stage of mast cell activation by the antigen, Syk, which is the most important signal transduction protein, activates various signaling substances, such as PLC*γ* and protein kinase C [19].

Lyn/Syk activation induces stimulation of MAPKs, which are involved in the production of proinflammatory mediators [20]. MAPK signaling plays an essential role in regulating the transcriptional activity of various cytokine genes in mast cells and is thus a major mechanism of treatment of allergic inflammation [21]. To elucidate the MAPK pathway of action of SIE on Fc*ε*RI-mediated allergic reactions in mast cells, we tested the effects of SIE on MAPK activation. Treatment with SIE reduces the phosphorylation of MAPK pathways, including ERK, JNK, and MEK1/2 genes in Ag/IgE-mediated RBL-2H3 mast cells. Additionally, it was found that SIE inhibited the phosphorylation of Akt and IkB*α* by Lyn/Syk activation [22]. These observations indicate that Lyn/Syk activation is essential for the degranulation signal transduction.

Next, we found that SIE inhibited COX-2 and cPLA2 expressions and reduced the levels of the PGD2 products, which are enhanced in the activated immune cells including mast cells. COX-2 and cPLA2 are pivotal enzymes for the production of proinflammatory lipid mediators and in the metabolism of arachidonic acid associated with allergic actions [13]. PGD2 is the major arachidonic acid metabolite secreted by mast cells and is known to be produced in response to allergic inflammation [23]. Transgenic mice overexpressing PGD synthase, with a resultant overproduction of PGD2, develop inflammation and Th2 cytokine response following allergen sensitization and exposure [24]. These findings suggest that SIE reduces Fc*ε*RI-mediated allergic reactions through the suppression of cPLA2 activation and inhibition of COX-2 activity.

Finally, we examined how SIE suppresses IgE-mediated PCA in mice. PCA is characterized by an immediate skin reaction at a localized IgE-mediated allergic response in vivo, typically with increased vascular leakage in the skin that can be assessed by an intravenous injection of Evans blue [25]. In vivo, PCA can be identified based on ear skin color and the number of mast cells in the allergic site at the ears. Consistent with in vitro findings, SIE successfully reduced allergic inflammatory responses in the PCA-induced mice. This result suggested that SIE inhibits IgE-mediated allergy responses by downregulating mast cell activation. Research based on natural products is associated with the development of complex drugs that influence multiple targets simultaneously, which may be superior in controlling complex disease systems, have lower drug resistance, and are standards of care in numerous important therapies.

## 5. Conclusions

This study showed the antiallergic effects of SIE against Fc*ε*RI-mediated mast cell activation through the downregulation of Fc*ε*RI and arachidonate expression, which might cause inhibition of degranulation in RBL-2H3 mast cells and antigen-IgE-mediated PCA reactions. Inhibition of the degranulation process results in reduced production of allergen mediators, such as histamine, TNF-*α*, IL-4, IL-6, and PGD_2_, caused by Ag/IgE interaction. These results indicate that SIE could be a beneficial treatment for allergy-related diseases.

## Data Availability

The data is not available. The data that has been used is confidential.

## Supplementary Materials

Supplementary Materials Method S1: chemicals and reagents. Method S2: HPLC analysis. Results S1: HPLC analysis of SIE. Figure S1: HPLC-DAD chromatograms.
