# Supplementary material for: Ethanol Extract of Sesamum indicum Linn. Inhibits FcεRI-Mediated Allergic Reaction via Regulation of Lyn/Syk and Fyn Signaling Pathways in Rat Basophilic Leukemic RBL-2H3 Mast Cells
Source: Mediators Inflamm. 2019 Oct 10;2019:5914396. doi: 10.1155/2019/5914396 (PMC6811790; doi:10.1155/2019/5914396)
Supplement: Supplementary Materials — Method S1: chemicals and reagents. Method S2: HPLC analysis. Results S1: HPLC analysis of SIE. Figure S1: HPLC-DAD chromatograms. [file 5914396.f1.pdf]

Supplementary method

S1. Chemicals and Reagents

Sesamin, (-)-asarinin and sesamolin were purchased from (Sigma-Aldrich Co. (St. Louis, MO, USA)). The purity of all reference standards was above 97%. HPLC-grade methanol was purchased form Fisher (Pittsburgh, PA, USA). The ultrapure water used for the HPLC analysis was prepared using Puris-Evo UP Water system with Evo-UP Dio VFT and Evo-ROP Dico20 (Mirae ST Co., Ltd., Anyang, Gyeonggi-do, Korea).

S2. High-performance liquid chromatography (HPLC) analysis

In order to identify three components in *Sesamum indicum* L. sample, the previously reported HPLC analytical literature was modified and referenced [S1-3]. In this study, HPLC analysis was processed using a Dionex UltiMate 3000 system (Dionex Corp., Sunnyvale, CA, USA) equipped with a binary pump, an auto-sampler, a column oven and a diode array UV/VIS detector (DAD). System control and data analysis were performed by Dionex Chromelon software. The components were separated on an Acclaim® 120 C18 column (150 × 4.6 mm, 5 μm, Dionex Corp., Sunnyvale, CA, USA), and the column oven temperature was kept at 40 ^{o}C. The three components were eluted in a gradient system consisted of solvent A (deionized water) and solvent B (methanol). The gradient elution system, to improve the chromatographic separation capacity, was programmed as follows: 30-65% B, 0-10 min; 65% B, 10-35 min; 65-80% B, 35-35.5 min; 80% B, 35.5-40 min; 80-30% B, 40-40.5 min; 30% B, 40.5-50 min at a flow rate of 0.8 mL/min. The injection capacity

was 5 μL, and all analytes were analyzed in three times repeatedly. The detection wavelengths for three components were set at 200, 230, 290 and 330 nm.

[References]

1. G-S Kim, D-H Kim, M-R Jeong, I-B Jang, K-B Shim, C-H Kang, S-E Lee, N-S Seong and K-S Song, Quantitative Analysis of Sesamin and Sesamolin in Various Cultivars of Sesame, Korean. J. Crop. Sci. 2004, 49(6), 496-502.

2. MJ Nam and HY Chung, Oxidative stability of sesame oil prepared from black sesame flour, Korean. J. Food. Sci. Technol. 2008, 40(2), 141-145.

3. HM Kim, JM Lee, JY Park, SL Lee and SH Lee, Determination of Sesamin in Sesame Dregs by High Performance Liquid Chromatography, Kor. J. Hort. Sci. Technol. 2008, 26(2), 197-201.

Supplementary result

The HPLC-DAD chromatograms of the three mixed standard components and the SI extracts were shown in Figure S1. Based on the UV spectra and the maximum absorption values of each of the standard components, the UV wavelength was optimized at 290 nm for the three compounds. Therefore, the 290-nm wavelength was selected as the wavelength for the HPLC analysis. Each peak of the three compounds in SI was identified based on their retention times (tR), UV spectra, and chromatogram patterns, against the respective values in the standards. All target components were well separated and displayed good selectivity in the standard compound mixture and no interference was observed from other components within 35 min. The retention times of each of the standard compounds were analyzed at 22.18 min (1, sesamin), 23.98 min (2, (-)-asarinin), and 27.35 min (3, sesamolin) in the chromatogram. Sesamin and sesamolin were detected with good sensitivity and selectivity in Sesamum indicum Linn under similar conditions. The retention times of the observed compounds were 22.16 min (1) and 27.32 min (3), respectively. However, (-)-asar inin (2) was not analyzed in the SI extract.

Supplementary Figure

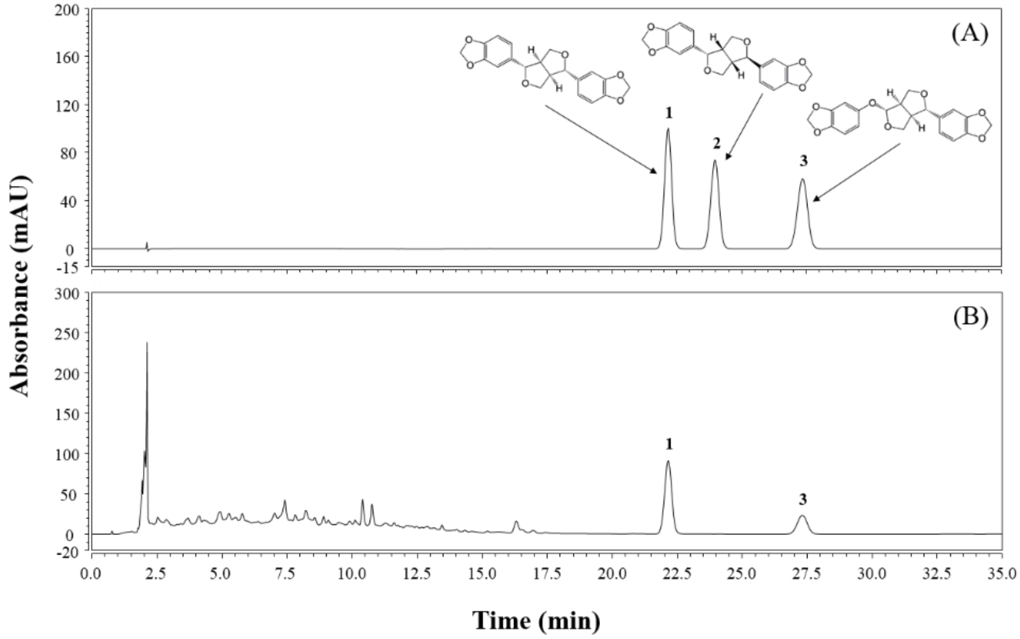

Figure S1. HPLC-DAD Chromatograms of the three standard compound mixture (A) and *Sesamum indicum* Linn. (B). Sesamin (1), (-)-asarinin (2) and sesamolin (3) were identified at 290 nm.
